# Healing in forgiveness: A discussion with Amanda Lindhout and Katherine Porterfield, PhD

**DOI:** 10.3402/ejpt.v5.24390

**Published:** 2014-09-01

**Authors:** Katherine A. Porterfield, Amanda Lindhout

**Affiliations:** 1 Department of Psychiatry, New York University Langone Medical Center, New York, NY, USA; 2 The Global Enrichment Foundation, Canmore, AB, Canada

**Keywords:** trauma recovery, resilience, forgiveness, trauma treatment

## Abstract

In 2008, Amanda Lindhout was kidnapped by a group of extremists while traveling as a freelance journalist in Somalia. She and a colleague were held captive for more than 15 months, released only after their families paid a ransom. In this interview, Amanda discusses her experiences in captivity and her ongoing recovery from this experience with Katherine Porterfield, Ph.D. a clinical psychologist at the Bellevue/NYU Program for Survivors of Torture. Specifically, Amanda describes the childhood experiences that shaped her thirst for travel and knowledge, the conditions of her kidnapping, and her experiences after she was released from captivity. Amanda outlines the techniques that she employed to survive in the early aftermath of her capture, and how these coping strategies changed as her captivity lengthened. She reflects on her transition home, her recovery process, and her experiences with mental health professionals. Amanda's insights provide an example of resilience in the face of severe, extended trauma to researchers, clinicians, and survivors alike. The article ends with an discussion of the ways that Amanda's coping strategies and recovery process are consistent with existing resilience literature. Amanda's experiences as a hostage, her astonishing struggle for physical and mental survival, and her life after being freed are documented in her book, co-authored with Sara Corbett, *A House in the Sky*.

Below, Amanda speaks about her recovery from her captivity in Somalia with Dr. Katherine Porterfield, a clinical psychologist at the Bellevue/NYU Program for Survivors of Torture.

*In* A House in the Sky, *you describe yourself before your kidnapping in Somalia as a young woman who was hungry to travel the world and make a difference in some way. For those of us who treat survivors of violence, it is essential to understand the whole person, not just the person who got hurt—the “victim.” Sometimes that means going back and awakening the part of the person that was present before these events, so that he or she can access strengths and the sense of self that is fundamentally still there, though changed in some profound ways. Tell me a bit about that early part of your life and where you think your desire to engage in the world and travel came from*.

From a young age I wanted to experience the world, but traveling wasn't something we did. My single mom worked as a cashier at a grocery store for little more than a minimum wage. My two brothers and I had to learn to fend for ourselves. After school, we were on the lookout for garbage dumpsters, which we'd climb into, hunting for discarded bottles that we could return for their deposit money. I took the coins I'd get to my favorite place, an old used bookstore, and I'd buy back issue copies of *National Geographic Magazine*, which I kept in a big stack beside my bed. Violent fights between my mother and her boyfriend often kept me awake at night, and some of my most vivid memories of childhood are of being huddled under a blanket with a flashlight and, tuning out the sounds of my house, escaping into the exotic and beautiful locales on the pages.

When I graduated from high school, university didn't seem like an option given my family's financial situation, so I moved to the nearest big city, Calgary, and started working. I got three jobs and saved all my extra money so I could purchase my very first plane ticket to Venezuela, a place straight out of the pages of my childhood magazines.

For the next 7 years, I traveled all over, India to Africa, the Middle East, South America, to over 50 countries. Between trips, I'd return home, working and saving to leave again; traveling fed an insatiable curiosity I had about cultures, places, and people. I was exploring spirituality, learning meditation, and reading books, such as *The Power of Now*, which would later become very valuable. The years passed, I was having adventure and fun, yet I deeply desired traveling that had purpose. I decided I wanted to be a journalist. I bought an expensive camera, took some photography classes, and based myself in Afghanistan and Iraq. I was teaching myself, and, though I didn't have much experience or training, I was beginning to have some small success. Eventually, I transitioned into television and worked as a freelance reporter for several stations.

As I traveled, I gained confidence in my ability to navigate the world. I was drawn to increasingly risky and dangerous places, and, bolstered by my newfound purpose of sharing stories as a journalist, into war zones. I focused on the human stories behind the war. I was drawn in, deeply, to human suffering. I witnessed many terrible things while living in Iraq, things I wish I could erase from my mind. I wanted, in some almost obsessive way, to understand what, in the mind and heart, enables human survival when so much has been destroyed. I was amazed to watch how life carries on in the midst of war. I wrote a weekly column in my hometown newspaper, and I'd often seek out survivors and tell their stories of war, AIDS, and hunger. During my short tenure as a reporter, and my years as a traveler, I was driven by my belief that the world is, at its essence, full of good people and kindness.

In 2008, I had been living in Baghdad for about 7 months and I had my eyes on Africa. Somalia, specifically, where there was an impending food crisis, war, and a humanitarian catastrophe. It was a country I had never been to and these were stories I believed were important to tell. I began planning a 1-week work trip to Somalia. I invited an Australian photographer, Nigel Brennan, to join me, and together we flew into Mogadishu.

On our fourth day in the country, the course of my life changed abruptly when my vehicle was ambushed by a group of gunmen, who abducted me and held me hostage for the next 460 days. In Somalia, my deepest beliefs about human goodness and connection, the beliefs that had always motivated me to be out in the world, were tested in the most unimaginable ways.

*When you were kidnapped, you were obviously unaware of how long your ordeal would last. Can you describe the conditions of your early captivity and whether you had particular strategies you used to survive the situation. Did those strategies change as the captivity lengthened?*

My first instinct, before it was even clear what exactly was happening, was to humanize myself to my captors. I recall introducing myself to the driver of the car as we sped away from the spot along the highway where they had abducted me, telling him that I am from Canada, about my time in the Middle East, and my respect for Islam. With every part of my being I wanted to live.

Nigel and I were taken to a house and shut inside a dark room together. We were both incredibly frightened and anticipating the worst. On the first day of captivity, I led Nigel through a meditation exercise I'd been practicing using the words, “I Choose Peace.” I taught him what I knew about deep breathing and meditation. Focusing on these skills was helpful for both of us.

Our captors were young jihadists. I'd been in Iraq long enough to learn a little about Islam, and I suspected that if we requested to convert to Islam, they would feel obligated to allow us. On the 11th day of captivity, Nigel and I went through the motions of becoming Muslim. We were given an English/Arabic Koran, taught to pray, and given new names. I was re-named Amina. My goal was to understand how their religion shaped the way they saw the world and I dived into reading the Koran. Using the Koran to have discussions with my captors, I was able to understand that they believed the abduction of two former “infidels” to be “by the right hand,” that our kidnapping was sanctified by Allah. I worked very hard to portray myself in a manner which I hoped would garner me some respect and humane treatment.

Nigel and I were separated from each other after 2 months. Suddenly alone, I became terrified of losing my mind. But I found I could keep a conversation going on within, and with, myself. I'd walk in circles all day, using my imagination to transport me out the room I was stuck inside. I'd imagine I was walking along the sea wall in Vancouver, running into friends. I would imagine every detail, how it would feel to be there, the sun on my face. As I walked, I told myself, “I'm one day closer to freedom.”

Everything changed after Nigel and I attempted an escape. We jumped out a bathroom window and ran to a nearby mosque, hoping we'd find help. But it didn't take our captors long to realize we were missing. We were recaptured and everything that followed was incredibly harsh punishment.

Chains were put around my ankles and I was locked up in a dark room, forbidden to sit up or lay on my back, I was confined to a dirty foam mat on the floor. During this period there was a great deal of violence, including sexual assaults and beatings. There was so much suffering and yet I didn't actively think about ending my life. Looking back is like recalling a dream – there is a slightly blurry quality and the days and months run together. I took solace in my mind, using imagination and memory to create another world to live in. In my mind, I went to school, I ate, I saw my family. In my mind, I could laugh at a joke, smile, see the sky.

When I was abused, it pulled me out of my imagination and back into the pain of my body. No matter how many times I was abused, I always felt the same things— disgust, hate, anger, rage— thick and powerful, like a poison inside my body, causing me physical pain. I knew at the deepest part of myself that feeling hate so deeply was not healthy. The rage I felt scared me, it felt alive inside of me. When my abuser would leave the room I would choose a mantra to say over and over, with controlled breathing, to calm down. I'd learned this, somewhere, years before. My mantras were often “I choose freedom,” “I choose peace,” and “I choose forgiveness.” I would say this over and over, for an hour or more, until my body felt calm. I was learning the power of the positive mind. Each day, I'd go through my body, head to toe and affirm my health and strength, even as my body suffered and starved. I had a clear vision in my mind of the healthy woman I wanted to one day become again.

*So, you're describing very powerful cognitive and mental processes you used to try to endure the suffering and uncertainty of your situation—visualization, breathing, self-talk, meditation. These are things that clinicians try to teach survivors of violence to use after living through a trauma. It strikes me that your ability to use them during your captivity may have really protected you and helped you to survive. Your ability to use your mind clearly protected you but also seemed to allow you to try to make meaning out of what was happening, rather than simply “live through it.” When did you find yourself developing a sense of understanding about your captors’ actions?*

I had many moments in captivity which I would call “moments of awakening” but the first, and most profound, came one day when I was being abused by the captor I hated most, Abdullah. Abdullah seemed to believe that he owned me and he took pleasure in hurting me. This day, I was feeling very fragile and, as he was abusing me, I had an experience of dissociation that completely changed the rest of my time in captivity. When I felt that internal “snap” and like I was out of body – suddenly observing in addition to experiencing [an observer in the room] – I was struck by an understanding of who the boy hurting me was and that his pain was at least equal to mine. I thought of the stories about his life that he had shared with me, of watching his neighbors killed, of finding a piece of his aunt's leg after the explosion that killed her. When I realized that he was driven to create suffering for me because of his own state of suffering, most unexpectedly, I felt compassion. I was confused by this feeling but it didn't go away. When the hate and anger would bubble up, I would touch that tiny seed of compassion and stay there because it felt like relief, it felt like the truth. The more I gravitated to compassion, the stronger my conviction to forgive became a gift to my own self, to save myself from the agony of hate. I developed a practice; each time I was hurt, I would try to release my anger by touching compassion.

Forgiveness was an active choice but not an easy one. No matter what they were doing to me, I still had the power to choose my response. I could choose to retain my own morals and values. That was the one thing over which I still had control. And so I would feel anger but I would make a choice to work through it and to let it go, for my own sanity. Sometimes I would argue with myself, wondering what the point of doing forgiveness work was, if they were going to continue to hurt me. I made that choice because I knew at the deepest part of my being that if I chose forgiveness I could not be defeated.

As time passed, I began to understand that I am not only my body, that there is spirit inside. My body was hurting but this part of myself was untouched. It was the voice that told me to hold on, to remember the beauty of the world, and to believe in freedom. This is the sense of self that comforted my panicked mind, saying, “You are ok.” This is one of the aspects that I find difficult to explain to others, the deep relationship I built with self. It was clearly, two selves, the suffering self and the compassionate self.

*You describe in your book how the young people who held you captive abused you. Do you think the impact on you of the interpersonal violence was different than the impact of other traumatic aspects of the captivity (isolation, lack of proper food/hygiene, lack of control over your surroundings, separation from loved ones, etc.) Because, in my experience as a clinician with survivors of violence, there is a particular struggle with how to get past the pain of that human cruelty—how to make sense of it afterwards. And that struggle requires a process of meaning making, in order to make sense of the cruelty*.

My darkest moments in captivity were not the times that the physical pain was the greatest, they were the times that my faith in human decency felt lost, when I began to question the inherent goodness of humankind, which I had always believed in so strongly. I could not understand how those young men, in each of whom I glimpsed moments of kindness, could also behave like they had no conscience at all. There was one point in captivity where everyone crossed a line, and I always knew the significance of that—of them all being part of it. It stopped any one of them from judging the others and unified them in my suffering. From that moment on they could not treat me as human being, for to do so would mean to confront their own actions to dehumanize me. Together, they slid into a very dark place.

I was in captivity for so long, with an up close look at the character of my captors, with a clear understanding that these young people were the products of war. My captors spoke about a desire for an education, to see the world, to live in a peaceful country. Yet, from the time they were born they were raised around guns and death. Everything they did to me was absolutely wrong; yet, I could understand how their lives had brought them to that point. Understanding helped me move closer to compassion, which, as the opposite of hate, felt better to me.

*When you were released and then returned home, after 15 months, describe a little bit about your mental and physical state. How did it change and develop over time? What kind of medical and mental health help did you receive or seek out?*

I had not been expecting to be released. That night, we were driven out into the desert and I was suddenly surrounded by men I did not recognize. I was sure that we had been sold to another gang, a threat they often made. It was only when someone put a phone to my ear and my mother was on the other end saying, “Amanda, you're free!” that I realized what was happening.

That night, in a hotel room in Mogadishu, I saw myself in a full-length mirror for the first time since I'd been captured. I hardly recognized myself. I was extremely thin, malnourished, and pale.

The next day I was flown to a hospital in Kenya, where I was reunited with my family. Freedom was hard to grasp. It felt like something I could lose again at any moment. Seeing the sky over head, my mother's smile, all of it felt capable of disappearing. I'd pinch my arm to make sure I wasn't dreaming. The dreams I'd had in captivity of freedom had been so vivid. Now that I was free, nightmares about captivity flooded my sleep. The line between dream and reality felt very blurry.

The Canadian government sent a psychologist to debrief me. Her plan was for me to tell my story, beginning to end, over the course of 10 days. She recorded the entire thing and then gave the tapes to the Royal Canadian Mounted Police and National Security for their ongoing investigation into my case. As I recall, the process felt forced. Since then, I have formally asked for those tapes to be destroyed, and, looking back, I find it insensitive that it was presumed that a long interview would be okay for me. Having just come out of an extended period without any control over decisions, I wasn't in a strong state of mind to refuse the interview, even though I wanted to. This can be a complicated aspect of someone's experience after a kidnapping—that is, the authorities' legal and forensic need for facts and details. It seems to me that this can be incredibly disruptive and hurtful to a person who has just been released and may not be able to process what they have been through. At least, that was my experience.

*You are describing what must have been a complete state of shock that you were in. The very mental processes that you used to protect yourself, while essential to your survival, were also dissociative in nature, meaning they allowed you to disconnect from the pain in your body and the horror of your circumstances. Those dissociative processes don't just “turn off” after a trauma. It sounds like you were living in that state between dissociation and reality in the aftermath of your release. The “debriefing” that you underwent would have been extremely threatening to your body and mind, as it forced you to relive events when you were not prepared to do so. Can you talk a bit about your recovery and your experiences seeking help from professionals once you were home?*

I was referred to several psychologists in the Calgary area, and I met with each of them. At the time, I was on the front page of all the newspapers and some of the psychologists showed familiarity with the media's interpretation of my “story” and that made me feel already judged. One day, I cold-called a male psychologist I found in the Yellow Pages, who then began working with me for almost a year. He had experience working with another former hostage, in South Africa, and he had not followed my story in the news. With him, I went through some, but nowhere nearall of, my kidnapping story and we focused a lot on reintegrating. During this time I felt happy to be home. I now know that the onset of posttraumatic stress disorder (PTSD) was delayed and that I was still actively “disconnecting” from my memories of what happened to me.

For the first 2 years after my release, I was getting out with friends, had enrolled in university and was future focused. After about a year, I stopped seeing the male psychologist, feeling like there was only so far I would be able to go with him, and began looking for a female therapist but, like the year before, I could not find anyone who felt like a good fit for me. Several therapists told me they did not have the expertise to work with a survivor of torture. Incredibly, none of the therapists I spoke with diagnosed me with PTSD.

*You began working with Sara Corbett, a writer who would become the co-author on your book, shortly after you returned. Tell us a bit about what it was like to describe the events of your captivity to your coauthor. Clinicians who read this journal will likely be wondering about whether the sharing of your narrative served a therapeutic function for you, as it does with many survivors of trauma. In therapy, the “exposure” to the narrative of the traumatic events is critical to recovery from post-traumatic difficulties. With narrative exposure, the survivor tells the story of the events they endured and is able to tolerate, with the therapist, previously intolerable memories and the physiological arousal they engender. Did you feel that this was what happened in your writing of the book? Because, what you did was not with a therapist and yet it was with a careful listener who then helped you give voice to your experience. That strikes me as incredibly therapeutic*.

When I came home I was contacted by writers, publishers, and film makers, people who wanted to tell my story. But I wasn't ready. A few months later, Robert Draper, a journalist friend, introduced me to Sara Corbett, an experienced journalist and author. I connected with Sara immediately and she and I began a long conversation about writing a book together, and then, over 3.5 years, creating *A House in the Sky*. We wanted the story to be much more comprehensive than just the captivity. We wanted to share the story of my early life and adventures as well. It worked so well because I trusted Sara implicitly. I told her everything, even the parts I knew I didn't want in the book, the parts I had never felt comfortable enough with a psychologist to reveal. She lived the story with me a million different ways. A chapter would go between us dozens of times, as we fleshed out details, colors, smells. It could be overwhelming and, often, I needed to step away. My publishers, agent, and Sara understood that this was a difficult process for me, and they gave me the space and time I needed. I also wonder if our process helped me see my life—and myself as a person—in the context of decades, over many countries, during times of happiness and freedom … and not just as a kidnapping survivor. We didn't isolate my personhood into that 460-day experience.

Sara and I would go away together, to beautiful and sunny places, to work on some of the dark passages. We traveled to Mexico and the Bahamas and, sitting on a beach, with a microphone fixed to my bathing suit, we'd talk. And as we talked about those events, I'd begin to process them. I felt utterly safe with Sara, and we were able to find the words to express the emotion of what I'd lived through. It was very confronting to try to pinpoint the emotion, the thoughts, the moments. My impulse was to push the memories away, but I took the book project very seriously, and because everything was so contained, just Sara and I, I'd allow myself to go to places that scared me.

*You've talked about a turning point for you, several years after your return home, where you went through a very difficult time and had to seek therapy again. Can you describe that?*

In April 2012, an audio recording of a phone call between my mother and me, during captivity, was put on the Internet. It was devastating to me and, I now know, was a trigger for the posttraumatic stress that had been hidden. After hearing it, I immediately became symptomatic. This was a very confusing time for me. Suddenly, I began experiencing severe dissociation, nightmares, hypervigilance, body aches, and pains, and depression. As I was not working regularly with a psychologist, I did not understand that this was PTSD.

I reached out to you at the Bellevue/NYU Program for Survivors of Torture and, in our first period of working together, you helped me to really understand posttraumatic stress and the way that it lives in the body and that it is highly physiological in nature. I came to believe, with you, that the audiotape had been a very visceral sensory reminder of one of the most terrifying times in my captivity. It was as if this sensory reminder opened the “flood gates” of other sensory memories. So, in our work together, just learning about that—learning that the sensory reminders activate the physiological fear arousal system and make you feel in danger again—was incredibly helpful.

*You and I began working together during this period and you received other psychological care as well. Can you talk about what was helpful to you and what were your biggest struggles?*

After the initial work where you and I identified my PTSD and you educated me about the condition a bit, it was clear that I needed to do some very focused work on my symptoms. I was referred to an intensive inpatient trauma recovery program. The daily classes included cognitive behavioral therapy (CBT), dialectical behavioral therapy (DBT), mindfulness, yoga, and dance. I had an active schedule and I learned a lot about coping with symptoms. I was curious about eye movement desensitization and reprocessing (EMDR), which I'd read so much about, and as part of my trauma treatment, I had three-hour sessions, three times a week, for 6 weeks. Unfortunately, EMDR made me depressed and sick and the effects would stay with me for days. I felt the sessions set me back from the gains I was making with other therapies.

After the inpatient work, I returned to working regularly with you and focusing on my most problematic symptoms—flashbacks, hypervigilance, dissociative episodes, and grief. Probably the most helpful work that we have done has been narrative exposure work, in which I revisited specific traumatic memories and processed them with you in detail over multiple sessions. This has been a catalyst for some very deep healing for me. Interestingly, the opportunity to read my book aloud for the taping of the audio book provided another opportunity to have an “exposure” to the events of my captivity. I spent 10 days in a small recording studio, alone, speaking, slowly and clearly. After that, I listened to it, 17 hours of my own voice, reading my own story, from the book I had co-authored, about the experiences which were so difficult. This was hugely therapeutic for me.

Talking about my feelings and symptoms is helpful. I use tools every day, such as visualization, self-talk, meditation, and grounding. Grounding is something that helps me with dissociative symptoms. I use several simple grounding techniques to feel my body in the present time, such as literally keeping my feet on the floor and reminding myself that I am safe. In addition to breathing and meditation, this year I have discovered that yoga is very effective in combating dissociation.

I also see a psychiatrist regularly who has helped me tremendously by prescribing medication that assists with my symptoms, particularly sleep problems.

There is another piece to treatment that has been helpful and that is recognizing that survivors of interpersonal violence sometimes carry around very negative attributions about themselves and can almost “attack” themselves with these negative self-thoughts. I have worked a lot on that, in terms of stopping negative, hurtful thoughts that I can sometimes feel about myself. Doing this with a trusted person is essential.

I still have my struggles—I am still afraid of the dark, loud noises make me jump and when a man gets too close, I want to run. But I do see that healing is taking place and I am learning to live with my trauma. I'm coming around to the idea that, while the kidnapping will always be a part of my life story and who I am, the trauma of it does not have to control me.

*What about speaking about these events in public? Most trauma survivors do not do that. What challenges does that present for you? What benefits do you gain from it, psychologically?*

Over the past 4 years, I have spoken a lot about aspects of my experience and what I have learned. I recall being surprised, at first, that people wanted to hear from me. I think I felt some pressure to speak and a therapist encouraged me to do it. At my first public events, I spoke more about the state of Somalia than I did about what had happened to me. I talked about my choice to forgive my captors because they are products of war. After one of these early talks, a man came up to me and told me that he was going to go home and call his father, whom he had not spoken to for many years, because he was finally ready to forgive him. His words motivated me to continue sharing my story.

I have come to enjoy public speaking and I absolutely believe in the messages of forgiveness, gratitude, and choice. But there are many days when I find it painful to give voice to those memories. I often cry when I am on stage. There are moments that will always hurt to talk about, but the more I speak about the story, the less effect it has on me. I measure what I share, with who, and how. I think this is important for survivors of trauma to have control over the telling of their stories. Even if someone is not doing public speaking about what happened to them, they still face times when someone might want to ask them about their traumatic experiences. I believe survivors should feel control over when and how much they talk about painful life events and should protect themselves from feeling “forced” or pressured in any way to share their story. Therapy can be a great place to think through this issue—that is, when and with whom a person wants to share their experiences.

*What message do you have for others who have experienced violence and trauma?*

I'd say don't be afraid to get help. It took years and years for me to find a therapist who could speak to me clearly about PTSD and symptoms. When I was triggered in 2012, I had no education about my condition. I felt very alone. The symptoms were severe and during that period, before I had any understanding of PTSD, it was extremely difficult to endure. The more I've learned about PTSD, the easier it's become to manage it. I try to educate people around me—parents, friends, supporters, and so on. I realize it's okay to say “I'm having symptoms” or “This is why I can't do X or Y … .” With the education I have received over the last year and a half, and the tools that I now have, I feel more hopeful than ever about recovery. And I love sharing that message with others who have been through trauma. The human spirit is amazingly resilient and I truly believe that the worst human hurt can be healed by connecting and sharing with other people, even when it seems so hard to revisit those painful memories.

## A Note from Dr Porterfield

It has been an honor to work with Amanda, as well as other survivors of trauma. Articles like this provide a unique opportunity for clinicians and researchers alike to learn about the remarkable resilience exhibited by survivors, like Amanda. Amanda said that she felt compelled to share human stories behind the war, to “understand what, in the mind and heart, enables human survival when so much has been destroyed.” In her eloquent discussion, she describes so clearly what qualities enable not only survival, but also the capacity to thrive in the face of trauma and severe adversity. Amanda has embodied both during and after her kidnapping several critical components of resilience, identified in the scientific literature, including optimism, cognitive flexibility, adaptive coping skills, social support, focus on physical well-being, and a personal moral compass (Iacoviello & Charney, 2014).

Specifically, I'd like to focus on what Iacoviello and Charney have termed, embracing a “personal moral compass.” For Amanda, her ability, and intentional decision, to feel compassion and forgiveness towards her captors in a situation of severe maltreatment was an especially critical capacity during and after her captivity. This compassion led Amanda to found the Global Enrichment Foundation (http://www.globalenrichmentfoundation.com/), a non-profit organization that engages educational and community-based empowerment programs to promote peace and development in Somalia. Her choice to continue to share her story here, through her book, and in public forums is an active one that stems from her values and beliefs about healing and preventing violence in the world through understanding its origins. Thus, I would argue that these acts—forgiveness, altruism and educating others—are part of Amanda's deeply moral response to the suffering she survived and witnessed.

Amanda's engagement in treatment provided her with coping skills that have also been linked to resilience after trauma. Particularly, her use of mindfulness, visualization, and self-talk—strategies that she naturally turned to during captivity—was strengthened in the treatment. Her attention to her physical well-being and health once she was released also facilitated her healing process. For survivors of violence and trauma, there are a range of empirically supported treatment options, including trauma-focused CBT, prolonged exposure, and EMDR (Ponniah & Hollon, 2009), and there is some promising early evidence supporting DBT, mindfulness, and adjunctive yoga in the context of trauma recovery (Bohus et al., 2013; Lang et al., 2012; Staples, Hamilton, & Uddo, 2013; Steil, Dyer, Priebe, Kleindienst, & Bohus, 2011). Some researchers have suggested mindfulness practices, such as the meditation exercises Amanda used throughout her captivity as well as since returning home, may be beneficial not only for treatment of PTSD, but also for the prevention of PTSD (Thompson, Arnkoff, & Glass, 2011).

As Amanda and I discussed the title for this article, I was impressed (though not surprised) by her decision to highlight “Healing in Forgiveness.” Although research has touched on the role of forgiveness, few studies have fully explored the role of forgiveness in treatment. Given Amanda's remarkable example, clinicians and researchers alike may be well served to further explore the ways that a survivor's active and empowering choice to move to a place of forgiveness may be central to resilience during and after trauma.
